# EFFICACY OF PULSED DYE LASER IN COSMETICALLY DISTRESSING FACIAL DERMATOSES IN SKIN TYPES IV AND V

**DOI:** 10.4103/0019-5154.44792

**Published:** 2008

**Authors:** Sujay Khandpur, Vinod K Sharma

**Affiliations:** *From the Department of Dermatology and Venereology, All India Institute of Medical Sciences, New Delhi, India*

**Keywords:** *Pulsed dye laser*, *verruca plana*, *angiofibromas*, *pyogenic granuloma*

## Abstract

**Background:**

Pulsed dye laser (PDL) has revolutionized treatment of vascular dermatoses. It has been successfully employed to treat several non-vascular conditions in fair skinned individuals without producing significant pigmentary and textural complications.

**Aim:**

A preliminary study was undertaken to assess its efficacy in cosmetically distressing facial, vascular and non-vascular dermatoses in Indian patients with skin types IV and V.

**Materials and Methods:**

Nine patients of ages 7 to 55 years, with facial verruca plana (VP- 4 cases), angiofibromas (AF- 4 cases) and multiple pyogenic granulomas (PG- one case) were recruited. They had no systemic complaints. Laser parameters used were (spot size/fluence/wavelength/pulse duration):VP- 5mm/5.5-7.5J/585nm/0.45ms; AF-5mm/6-8.5J/585nm/0.45ms;PG- 5mm/7J/585 and 595nm alternately/1.5ms. Response was assessed clinically and photographically.

**Results and Conclusions:**

All VP lesions completely resolved after 2-4 sessions (mean 3.25 sessions), AF showed 50% regression in all cases after 2-3 sessions (mean 2.5 sessions) and ≥75% subsidence after 3-7 sessions (mean 5.5 sessions) and in PG, after 3 sessions, there was complete subsidence of small satellite lesions with moderate shrinkage of larger papules and complete resolution after 5 sessions. Complications included transient hyperpigmentation/hypopigmentation only. There was no recurrence during next 6 months. PDL offers significant cosmetic improvement in facial dermatoses in Indian patients.

## Introduction

Pulsed dye laser (PDL) has revolutionized the treatment of vascular dermatoses. In fact, it is the treatment of choice for port-wine stains and has shown good to excellent cosmetic results in facial lesions in patients of all skin types.^1^,^2^ It is also being widely used to treat several nonvascular dermatoses because of low complication profile including pigmentary and textural alterations.^3^,^4^ Facial dermatoses are a cause of tremendous cosmetic concern and produce severe psychosocial impact. PDL has been shown to be beneficial in these conditions in fair-skinned (skin types I–III) individuals. We undertook this study to assess the cosmetic results in various facial dermatoses in Indian patients with skin types IV and V.

## Materials and Methods

A total of 9 cases (3 males, 6 females) with skin types IV and V and age ranging from 7 to 55 years and with various facial lesions that produced significant psychosocial distress were inducted. They included 4 patients with multiple verruca plana (VP), 4 with multiple angiofibromas (AF) and one case with several, 1 mm–2 cm-sized pyogenic granulomas (PG), present on the left cheek. No previous intervention had been undertaken in any of the AF and in 3 of 4 VP patients. Electrocautery in one VP patient and surgical excision in PG was followed by recurrence. None of the patients were immunosuppressed. There was no systemic abnormality except in two AF cases where calcified subependymal nodules were detected on CT scan of the head. ELISA for HIV was negative.

Treatment with the flashlamp pulsed tunable dye laser (Cynosure VLS, Chelmsford, Massachusetts, USA) was initiated. History of herpes infection was excluded prior to therapy. The lesional site was occluded with EMLA cream (2.5% lignocaine + 2.5% prilocaine) for 45 min prior to the procedure. The laser parameters used were: for VP: spot size – 5 mm, fluence – 5.5–7.5 J/cm^2^, wavelength – 585 nm, pulse duration – 0.45 ms; for AF: spot size – 5 mm, fluence – 6–8.5 J/cm^2^, wavelength – 585 nm, pulse duration – 0.45 ms; and for PG: spot size – 5 mm, fluence – 7 J/cm2, wavelength – 585 and 595 nm alternately, pulse duration – 1.5 ms. In VP, single pulses per lesion at purpuric threshold were delivered; in AF, two pulses per lesion were administered, while for PG, 4–5 pulses per lesion were administered after compressing the lesions with a glass slide. Therapy was delivered in combination with continuous air cooling (Cryo 5, Zimmer Elektromedizin GmbH, Neu-Ulm, Germany). The procedure was repeated 4–6 weekly. Postoperatively, the patients were advised strict sun protection with application of high-SPF (34) sunscreen during the day and steroid-antibiotic cream at night. In PG, the lesions developed black eschar after each session. The patients were assessed both clinically and photographically at each session.

## Results

**Fig. 1:**
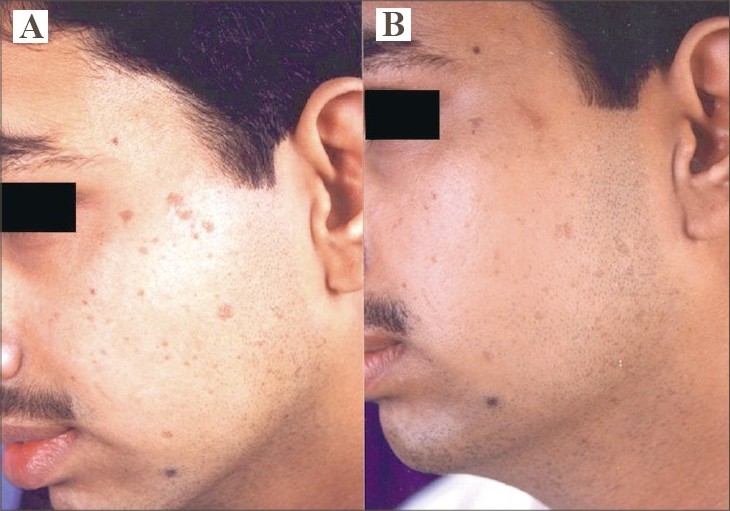
(A) Multiple VP over left side of face. (B) Complete resolution of all lesions with hyperpigmentation after 4 sessions.

**Fig. 2:**
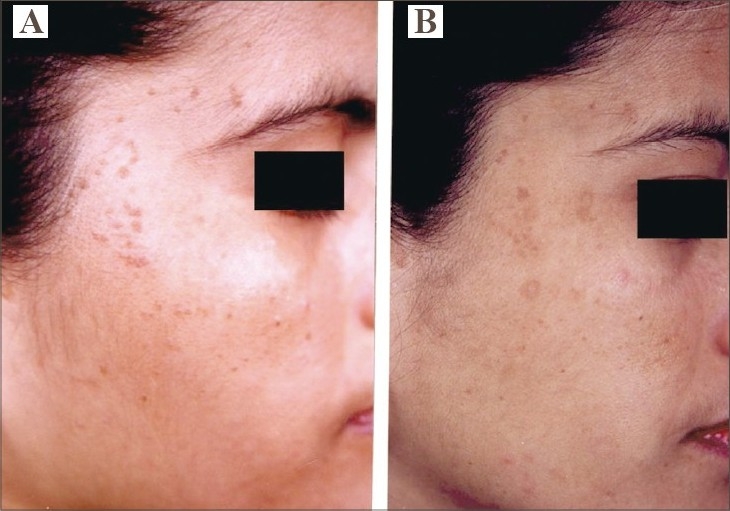
(A) Multiple VP over right side of face. (B) Complete resolution of all lesions with hyperpigmentation after 2 sessions.

**Fig. 3:**
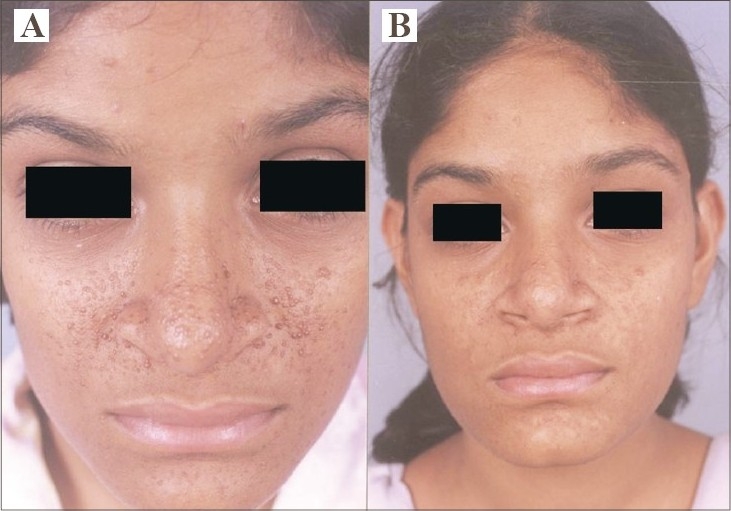
(A) Multiple AF of the face. (B) >75% reduction of lesions after 6 sessions with mild hypopigmentation.

**Fig. 4:**
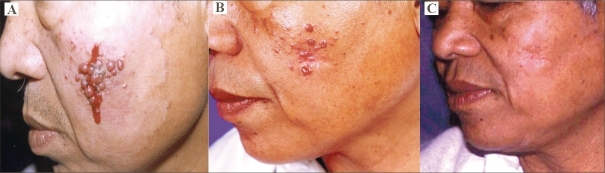
(A) Multiple 1 mm–2 cm-sized PGs over left cheek. (B) Complete resolution of small lesions and partial shrinkage of larger papules after 3 sessions. (C) Complete subsidence of all PGs after 5 sessions.

All VP lesions resolved completely after 2–4 sessions (mean 3.25 sessions). In AF, there was 50% regression in all cases after 2–3 sessions (mean 2.5 sessions) and >75% subsidence after 3–7 sessions (mean 5.5 sessions). In PG, after 3 sessions, there was complete subsidence of the small satellite lesions with moderate shrinkage of the larger papules. All lesions resolved after 5 sessions. All VP cases and 3 of 4 AF cases developed mild hyperpigmentation while one AF case developed hypopigmentation. There was no textural change. No recurrence was observed in any of the patients during the next 6 months. (Figs. 1–4)

## Discussion

A preliminary study is undertaken to assess the efficacy of PDL in Indian patients for the first time in various facial disorders that were both psychologically and physically distressing due to their rapid spreadability, interference with shaving or use of make-up or excessive bleeding on slight manipulation.

Previous studies have shown excellent cosmetic response in verrucae involving the face, back and proximal extremities within^1^–^3^ PDL sessions, probably due to specific destruction of superficially dilated capillaries and killing of virally infected cells by nonspecific thermal damage to the keratinocytes or by stimulating local cell-mediated immunity.^5^,^6^ Verrucae at these sites, by virtue of being present on soft, more pliable surface, show better response than palmoplantar or periungual warts. In all our patients, VP lesions resolved after a mean of 3.25 sessions.

The cure rate with PDL has been found comparable to conventional modalities (cure rate: 56%–80%) such as topical cauterizers, electro- and cryotherapy, surgical excision and lasers such as CO_2_ and KTP-Nd:YAG laser.^7^ However, it carries the advantage of a low complication profile, better response in recalcitrant lesions, low recurrence rate and better compliance, since it is delivered only once in 3–4 weeks. In our VP patients, mild and transient hyperpigmentation was the only side-effect and there was no recurrence. Vargas *et al*. did not observe any recurrence in any of their 12 facial verruca cases treated with PDL.^5^

Facial AF, especially if large and extensive, causes considerable disfigurement and emotional distress, obstruction of vision and hemorrhage when traumatized, requiring prompt intervention. Conventional modalities such as electro- or cryotherapy, dermabrasion, excision and lasers such as the continuous wave CO_2_, argon and copper vapor lasers have shown only moderate cosmetic results and several complications.^8^–^10^ In one study, continuous wave and super pulsed CO_2_ laser produced hypertrophic scarring in 23% cases necessitating treatment with intralesional steroids.^3^ PDL has been shown to be a useful option, especially for AF with a predominantly vascular component. Papadavid *et al*. showed excellent response in 92% of their facial AF lesions with a predominantly vascular component after mean of 2.83 sessions.^3^ Two cases required 6 sessions, while in 3 cases, additional electrocautery was required. In large AFs with both vascular and fibrous components, combined use of CO_2_ and PDL have shown greater benefit. All our facial AF cases showed excellent result (>75% flattening) after a mean of 5.5 sessions with only mild pigmentary changes and no scarring or recurrence.

We also achieved complete resolution of multiple and recalcitrant PG using two wavelengths (585 and 595 nm) alternately, without any side-effects or recurrence. Conventional procedures such as surgical excision, cautery (thermal or chemical), curettage and cryotherapy have shown high recurrence rates (approximately 43.5% reported after excision and cautery).^11^,^12^ They also produce hypo- or hyperpigmentation and pock-like or linear scars. Various laser systems including the continuous and pulsed CO_2_, argon, KTP, Nd:YAG and single wavelength (585 nm) PDL have also been tried with side-effects similar to the conventional modalities.^13^–^15^ The 585 nm/ 450∝s PDL cannot ablate the deeply located, larger caliber vessels in large polypoidal types of PGs.

Tay *et al*. reported recurrence in 9% of their patients, especially those with large or pedunculated lesions, following treatment with the 585 nm PDL.^16^ They could achieve complete resolution only in PG with a diameter less than 5 mm. Gonzales *et al*., however, demonstrated both symptomatic and clinical clearing of the lesions with excellent cosmetic results in 16 of 18 treated patients.^17^ The tunable PDL carries the advantage of emitting radiation over a wide spectrum of wavelengths (585 to 600 nm) and pulse durations (short and long). The longer wavelengths penetrate deeper and the larger pulse duration causes more even heating of larger vessels. In our patient, due to the large size of lesions on the face, alternate use of 585/595 nm along with longer pulse duration led to complete resolution after 5 sessions.

The tunable PDL was found to be a useful modality in the treatment of facial VP, AF and PG in Indian skin types. Large-scale studies are necessary to further establish its efficacy in these conditions.
